# Effects of a training program after surgically treated ankle fracture: a prospective randomised controlled trial

**DOI:** 10.1186/1471-2474-10-118

**Published:** 2009-09-25

**Authors:** Gertrud M Nilsson, Kjell Jonsson, Charlotte S Ekdahl, Magnus Eneroth

**Affiliations:** 1 Department of Health Sciences, Division of Physical Therapy, Lund University, Lund, Sweden; 2 Department of Radiology, Lund University Hospital, Lund, Sweden; 3 Department of Orthopaedics, Malmö University Hospital, Malmö, Sweden

## Abstract

**Background:**

Despite conflicting results after surgically treated ankle fractures few studies have evaluated the effects of different types of training programs performed after plaster removal. The aim of this study was to evaluate the effects of a 12-week standardised but individually suited training program (training group) versus usual care (control group) after plaster removal in adults with surgically treated ankle fractures.

**Methods:**

In total, 110 men and women, 18-64 years of age, with surgically treated ankle fracture were included and randomised to either a 12-week training program or to a control group. Six and twelve months after the injury the subjects were examined by the same physiotherapist who was blinded to the treatment group. The main outcome measure was the Olerud-Molander Ankle Score (OMAS) which rates symptoms and subjectively scored function. Secondary outcome measures were: quality of life (SF-36), timed walking tests, ankle mobility tests, muscle strength tests and radiological status.

**Results:**

52 patients were randomised to the training group and 58 to the control group. Five patients dropped out before the six-month follow-up resulting in 50 patients in the training group and 55 in the control group. Nine patients dropped out between the six- and twelve-month follow-up resulting in 48 patients in both groups. When analysing the results in a mixed model analysis on repeated measures including interaction between age-group and treatment effect the training group demonstrated significantly improved results compared to the control group in subjects younger than 40 years of age regarding OMAS (p = 0.028), muscle strength in the plantar flexors (p = 0.029) and dorsiflexors (p = 0.030).

**Conclusion:**

The results of this study suggest that when adjusting for interaction between age-group and treatment effect the training model employed in this study was superior to usual care in patients under the age of 40. However, as only three out of nine outcome measures showed a difference, the beneficial effect from an additional standardised individually suited training program can be expected to be limited. There is need for further studies to elucidate how a training program should be designed to increase and optimise function in patients middle-aged or older.

**Trial Registration:**

Current Controlled Trials ACTRN12609000327280

## Background

Ankle fractures are among the most common fractures in the lower extremity [1,2]. In younger ages the incidence is higher among men but at the age of 50 the gender ratio reverses [1,3]. The incidence rate has been reported to be between 101/10^5 ^person years [2] to 107/10^5 ^[3]. Dislocated fractures and fractures resulting in instability of the ankle mortise are surgically treated with internal fixation using screws and plates and/or cerclage, staples and pins [4,5]. Most fractures are immobilised in a plaster cast for between six and eight weeks [6-9].

The results after surgically treated ankle fractures are conflicting. Evaluating subjective recovery using the Olerud-Molander Ankle Score (OMAS) some studies have reported only slight disability [7,10-12] whereas more recent reports have shown poorer results [13,14]. Using the OMAS Nilsson et al. showed in subjects over the age of 40 years a risk of subjectively scored poor function [15]. Similar results were found by Egol et al. using the Short Muscular Function Assessment (SMFA) [16]. Regarding physical outcomes, decreased balance capacity and ankle joint mobility were found to be independently associated with symptoms and poorly scored function one year after injury [17].

Neuromuscular training programs are commonly used in clinical practice for lower extremity rehabilitation. These programs are mainly applied in rehabilitation after knee injuries [18] and after ankle ligament injuries [19-21]. There are a limited number of studies that have evaluated the results of a rehabilitation program after surgically treated ankle fracture [8,22]. Shaffer et al. showed normalised muscle performance, functional ability and fatigue resistance following ten weeks of physical therapy focusing on strengthening and ambulation. The study included ten patients mean 35 years and the results were compared to an age-and gender matched control group [8]. Stevens et al. found similar results in a group of nine individuals mean 21 years [22]. To our knowledge no randomised controlled trials have been performed evaluating the effect of a standardised training program after plaster removal.

The aim of this randomised controlled study was to evaluate if a standardised but individually suited training program, supervised by a physiotherapist, starting within one week after plaster removal, focusing on regained ankle joint mobility, muscle strength, balance and functional training could improve symptoms, subjectively scored and physical outcome in patients 18-64 years of age compared to usual care.

## Methods

### Study design and setting

**Figure 1:**
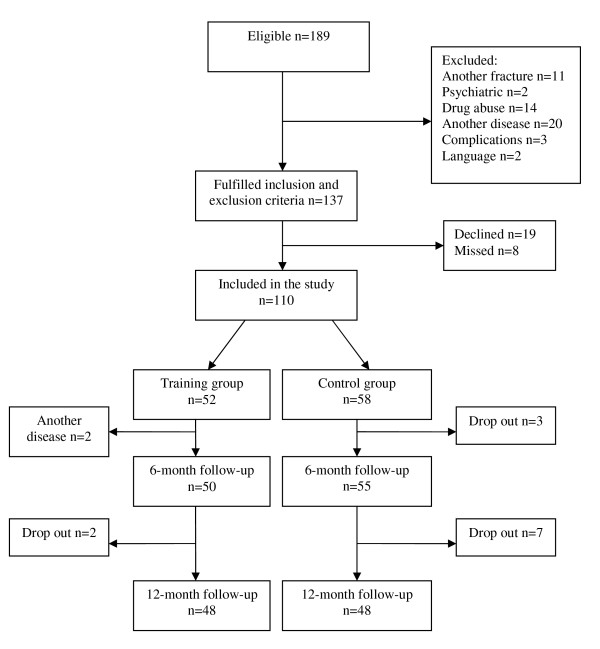
**Flow chart and randomisation process of the patients included in the study**.

This is a prospective, randomised controlled trial performed at the Department of Orthopaedics, University Hospital, Lund, Sweden in collaboration with physiotherapists in primary health care. During January 2003 until October 2005, consecutive patients with a surgically treated ankle fracture, 18-64 years of age and living within an area of 50 kilometres from the University Hospital, Lund, Sweden were eligible for inclusion. One hundred-eighty-nine patients fulfilled these criteria. Exclusion criteria were diseases that might influence the physical function of the lower extremity or adherence to the randomisation process: co-existing fracture on the other leg or another fracture on the same leg (n = 11), psychiatric diagnosis (n = 2), drug abuse (n = 14), symptomatic osteoarthritis in the lower extremity, rheumatic or other systemic diseases (n = 20) delayed surgery due to complications (n = 3) and persons not proficient in the Swedish language (n = 2). Thus 137 fulfilled the inclusion criteria for this study. Of these, 19 declined participation and eight were missed for inclusion during their hospital stay (14 men aged (median) 32 and 13 women aged (median) 52) resulting in 110 participants. Of these 52 were allocated to the training group and 58 to the control group. Before the first follow-up at six months after the injury two patients allocated to the training group were excluded secondary to a new injury in the same ankle (n = 1) and a cerebral haemorrhage (n = 1) and additionally three patients allocated to the control group dropped out (declined to participate) resulting in 105 subjects left for the study (Figure 1).

The day after surgery patients were given written and verbal information about the study and were asked to participate in the study. After written informed consent, the patients were randomised by sealed envelopes allocating them either to a standardised rehabilitation program (training group) or to usual care (control group). All subjects had to complete a questionnaire at six and 12 months after injury and were examined by the same physiotherapist (GN) who was blinded to the allocation group. At the 12-month follow-up the patients were also examined by a doctor for a medical check up and radiological examination. The Research Ethics Committee at the Lund University approved the study (LU297-02)

### Patients and fracture characteristics

Study data are presented for the 105 patients. The mean age for men was 37 (SD 13) years and for women 48 (SD 12) years (p < 0.001). Age, height, weight and BMI did not differ between the two random groups (Table 1). The left ankle was injured in 60 (57%) patients, 96 injuries (91%) were falls on level ground or in on stairs, four were bicycle accidents, two were motorbike accidents and three were falls from a height. Twenty-two patients had been injured during sport/leisure time activities.

**Table 1:** Patient characteristics in subjects with surgically treated ankle fractures (mean, SD)

**Variables**	**Training group**	**Control group**	**p-value^¤^**
	**Mean (SD)**	**Mean (SD)**	
	**n = 50**	**n = 55**	
Age (years)*			
- men (n = 43)	34 (22)	32 (26)	0.751
- women (n = 62)	51 (22)	51 (19)	0.559

**Height (cm)#**			
- men	180 (6)	179 (7)	0.674
- women	166 (7)	167 (7)	0.629

**Weight (kg)#**			
- men	87 (13)	91 (18)	0.379
- women	78 (16)	75 (17)	0.418

**BMI (kg/m^2^)#**			
- men	27 (3)	28 (5)	0.186
- women	28 (5)	27 (4)	0.191

*At injury; ^#^At 6-month follow-up; BMI = Body Mass Index; ^¤^Independent Student *t*-test

The same radiologist (KJ) examined all the images and the fractures were classified according to Lauge-Hansen [23] showing 80 supination injuries (3 SA II, 1 SE I, 9 SE II, 1 SE III, 66 SE IV) and 25 pronation injuries (2 PA II, 9 PE III, 14 PE IV). Sixty patients (57%) were surgically treated with the method described by Wiberg-Cedell [4] using cerclage, staples and/or pins. Eighteen (17%) patients were treated with the method described by AO group (in German: Schweizerischen Arbeitsgemeinschaft für Osteosynthesfragen) [5] using plate and screws and 27 (26%) with a combination of these two methods. Sixty-four (61%) patients underwent uni-malleolar fixation, 37 (35%) had bi-malleolar and 4 (4%) tri-malleolar fixation. The medial malleolus was only treated when fractured. The posterior margin of tibia was fractured in 65 (62%) cases of which eight were fixated by one or two screws. The remaining fractures of the posterior margin of the tibia comprised less than 25% of the articular surface with minimal displacement and were judged not to be in need of fixation. Radiographic examination including ankle joint congruency, fracture healing, fracture reduction and presence of osteoarthritis (loss of joint space less than 50%; loss of joint space more than 50% but no bone-to-bone contact; bone-to-bone contact) was performed pre-surgery, post-surgery and at 12-month follow-up. The post-operative radiographic results showed complete ankle joint congruency in 103 (98%) ankles and less than 2 mm in-congruency in two. Post surgery fracture reduction was complete in 53 (50%) ankles, < 2 mm in-complete in 33 (31%) ankles, ≥ 2 mm in-complete in 18 subjects (17%) and one was a proximal fibula fracture.

All but one patient were given a below knee plaster cast and the plaster time was a mean 43 days (SD 5.5). Three patients had superficial wound infection, one had a small local skin necrosis, and two had a deep vein thrombosis. The mean number of days spent in hospital was 3.6 (SD 1.6) days. During the first six weeks in plaster 81 (77%) were prescribed non-weight bearing gait. The remaining patients were allowed partial weight bearing. All fractures were classified as clinically healed at the final plaster removal. Twenty-three patients had another surgical treatment on the same fracture within the time of the study; two due to insufficient stability of the fracture, five due to extirpation of the syndesmotic screw before weight bearing and 16 were re-operated after fracture healing due to symptoms of the osteosynthetic material. Patient and fracture characteristics are described by allocation groups with no differences found between groups (Table 2).

**Table 2:** Baseline data in subjects with surgically treated ankle fractures

**Variables**	**Training group** **n = 50**	**Control group** **n = 55**	**p-value**
**Gender**			0.557^a^
- men	19	24	
- women	31	31	

**Injured side**			0.573a^a^
- left	30	30	
- right	20	25	

**Type of fracture**			0.111^a^
- supination	42	38	
- pronation	8	17	

**Internal fixation**			0.408^a^
- uni-malleolar	28	36	
- bi-malleolar	19	18	
- tri-malleolar	3	1	

**Operation technique**			0.559^a^
- Wiberg-Cedell	26	34	
- AO	9	9	
- Mixed technique	15	12	

**Joint congruency post surgery**			1.000^b^
- complete	49	54	
- ≤ 2 mm in-congruency	1	1	

**Reduction results post surgery**			0.390^a^
- complete	29	25	
- ≤ 2 mm displacement	15	18	
- > 2 mm displacement	6	12	

**Complications**			0.604^a^
- no complications	46	54	
- superficial infection	2	1	
- deep vein thrombosis	2	0	

**Sport before injury**			0.887^a^
- yes	36	39	
- no	13	15	
- missing information	1	1	

**Activity level before injury**			0.169^c^
- walking (2)	5/36	6/39	
- long walking (3)	8/36	14/39	
- jogging 2 times/week plain ground (4)	12/36	14/39	
- jogging cross-country or aerobics 2 times/week 5)	7/36	3/39	
- jogging 5 times/week (6)	5/36	2/39	

^a ^Chi-Square test; ^b ^Fisher's Exact test; ^c ^Mann-Whitney *U*-test

### Training program

Patients in the training group were referred to a physiotherapist in primary care who had accepted to participate and had received verbal and written instructions about all aspects of the study. The training started within one week after plaster removal and continued for twelve weeks with two appointments per week. Between the appointments the patients had to perform home exercises daily prescribed by the physiotherapist, appropriate to the functional status at the time. The training program was based on neuromuscular principles, standardised, following a certain progress during the twelve weeks (Additional File 1). As soon as one level of function was reached the training demands were increased. Functional goals were set up for loaded ankle dorsiflexion (30°) [6,24], plantar flexion (45°) [6], one-leg-stance 60s [25], rising on toes (n = 25; women over 50 years of age n = 20) [26,27], rising on heels (n = 20), normalised walking pattern when walking on even ground and on stairs and walking at comfortable speed 30 m (20 s) [8]. If the functional goals were met before the twelve week period the patient could be released at the earliest after eight weeks but had to be re-examined in week ten and twelve to assure that the achieved function was maintained.

### Control group

The control group followed usual care after plaster removal. That is instructions from the physician to start walking and return to normal function as soon as possible. Furthermore, based on the individual doctor's judgement a referral to a physiotherapist was in some cases given. Patients in the control group were also free to seek physiotherapy if they chose. The standardised training program aimed for the training group was not disclosed for the usual care group. That is as soon as a physiotherapist was included in the study and had taken part of the specific training program a written consent had to be returned to the project leader (GN) that no other patients with surgically treated ankle fractures than those included in the study and directed to her were trained by her until the study was finished. The physiotherapist was also instructed not to inform colleagues, who might treat patients in the usual care group, about the program.

### Age-groups

Forty individuals were younger than 40 years of age, 20 of these were randomised to the training group and 20 to the control group. Sixty-five were over 40 years of age, 30 of these were randomised to the training group and 35 to the control group (p = 0.53).

### Outcomes

#### Olerud-Molander Ankle Score

The Olerud-Molander Ankle Score (OMAS) [28] was the primary outcome measure and is a self-administered patient questionnaire. The scale is a rating scale from 0 (totally impaired) to 100 (completely unimpaired) and is based on nine items: pain, stiffness, swelling, stair climbing, running, jumping, squatting, supports and work/activities of daily living. The OMAS has been frequently used to evaluate symptoms and subjectively scored function after ankle fracture [10,12,28,29]. The score is validated against (a) linear analogue scale (LAS) measuring subjective recovery, (b) range of motion in loaded dorsiflexion, (c) presence of osteoarthritis and (d) presence of dislocations on radiographs, and it has been found to correlate well with these four parameters [28]. No floor or ceiling effect has been reported [30].

#### Short-form SF-36

The Short-form 36 (SF-36) is a self-administered generic questionnaire designed to evaluate health-related quality of life. The instrument measures eight health domains using eight scales assessing physical function (PF), role limitation due to physical problems (RP), bodily pain (BP), general health (GH), vitality (VT), social function (SF), role limitation due to emotional problems (RE), and mental health (MH). Low score implies poor health status high score implies good health status [31]. The SF-36 has been validated for use in Sweden and normative data on healthy people have been reported [32]. No studies evaluating reliability and validity of SF-36 for use in ankle fractures have been found. However, the SF-36 has been employed in some studies concerning patients with ankle fractures [12,33-36]. The summary scales of Physical Health (PCS) and Mental Health (MCS) were analysed.

#### Functional measures

Ambulation was assessed using the timed 9-meters walking test performed at the maximum speed [8]. Furthermore, the timed stair-climbing test was performed, that is the time to ascend a flight of stairs (12 steps) using a self-selected technique. If possible the test should be performed without using a handrail [8].

#### Physical examination

Lower limb muscle strength was evaluated using rising on toes and heels. Ankle mobility was examined in loaded dorsiflexion and plantar flexion [17]. Loaded dorsiflexion was measured with the technique described by Lindsjö et al. [24]. The head of the fibula was marked. The patient stood with the examined foot placed on a stool about 30 cm high. With the sole of the foot flat on the stool the patient leaned forward with the greater part of the body weight on the examined foot to the point where the heel was just in contact with the stool surface. The angle between the stool surface and a line going through the tip of the lateral malleolus and the mark at the head of the fibula was measured using a standard goniometer. The normal value for dorsal extension was stated as 30° [37]. To measure plantar flexion the patient sat on the edge of a stool. The leg investigated was stretched forwards with the whole sole of the foot on the floor to the point where the medial part of the forefoot was just in contact with the floor. The angle between the floor and a line going through the tip of the lateral malleolus and a mark at the head of the fibula was measured using a standard goniometer. Measurements were carried out on both sides.

### Statistical analysis

The statistical tests were performed according to the intention to treat principles. Sample size calculation regarding the main outcome measure OMAS showed that a sample size of 51 individuals per group was needed to detect a difference of 10 points between groups with an alfa level of 0.05 and 80% power. We performed mixed model analysis on repeated measures of OMAS, the sub-scores PCS and MCS of SF-36, the muscle strength tests and the timed walking tests as dependant variables, and treatment group, gender, age-group, follow-up time as fixed factors and the subjects as random factors. Analyses were performed both with and without interaction between age-group and treatment effect. The correlation between residuals within individuals was modelled as autoregressive (AR (1)). As an age younger than 40 years has been found as a predictor of recovery after surgically treated ankle fractures the patients were divided in two groups over or younger than forty years of age [16]. When analysing differences between groups regarding baseline characteristics, baseline data and radiological results the Student *t*-test, Mann-Whitney *U*-test and Chi-square tests were employed. An alpha level of 0.05 was regarded as statistically significant.

## Results

### Adherence to the program

All subjects remained in their allocation group throughout the study. The training group had a mean of 17 (SD 6) appointments with the physiotherapist and the control group had a mean of 7 (SD 8) (p < 0.001). Thirteen patients in the control group did not visit a physiotherapist. Out of these all but two patients described different activities that they had performed on their own in order to regain function such as increased daily walking, movements of the ankle regularly, dancing, aerobics, balance training, strength training and stretching.

### Olerud-Molander Ankle Score

Subjects younger than 40 years in the training group scored higher OMAS than those who were over 40 at both follow-ups. When including the variables age-group, gender, treatment group and follow-up time in the mixed model analysis no differences were found between groups (p = 0.310). When also including the interaction between age-group and treatment effect in the model a significant difference was found in favour of the training group in subjects younger than 40 (p = 0.028) (Table 3).

**Table 3:** Results from OMAS and SF-36 (mean (SD)) 6 and 12 months after surgically treated ankle fractures

**Variables**	**6-month** **Training group**	**6-month** **Control group**	**12-month** **Training group**	**12-month** **Control group**	**P-value#**
**OMAS (0-100)**					**0.028**
- all	62.4 (25.1)	63.5 (20.9)	74.4 (19.7)	71.4 (22.3)	
- <40 years	78.1 (15.7)	65.5 (15.4)	86.5 (12.4)	72.8 (17.6)	
- ≥40 years	51.4 (24.9)	62.4 (23.4)	67.5 (20.0)	70.7 (24.6)	

**SF-36 Physical health (PSC)**					0.273

- all	45.4 (10.6)	43.7 (11.1)	46.8 (10.3)	46.4 (9.4)	
- <40 years	50.8 (9.0)	42.0 (10.5)	51.1 (9.0)	49.2 (6.2)	
- ≥= 40 years	41.4 (10.0)	44.5 (11.4)	44.0 (10.31)	45.0 (10.6)	

**SF-36 Mental health (MCS)**					0.753
- all	45.3 (13.3)	49.4 (12.4)	48.3 (10.6)	51.2 (9.8)	
- <40 years	46.0 (14.0)	52.8 (10.5)	51.3 (9.3)	54.0 (5.5)	
- ≥40 years	44.9 (13.0)	47.7 (13.0)	46.5 (11.0)	50.0 (11.2)	

OMAS = Olerud-Molander Ankle Score; PCS = Physical Health Score; MCS = Mental Health Score

#Mixed model analyses on repeated measures for differences between training group and control group and change over time adjusting for gender, age-group, follow-up time and interaction between age-group and treatment effect. The p-values reflect the interaction between age-group and treatment effect.

### Short-form SF-36

The Summary Scale of Physical Health (PCS) showed higher scores at 6-month in the training group in patients younger than 40 years. However, when including the variables age-group, gender, treatment group and follow-up time in the mixed model analysis no differences were found between groups (p = 0.554). Neither when including the interaction of age-group and treatment effect in the model was any significant difference found (p = 0.273) (Table 3). Neither in the Summary Scale of MCS was any difference found between the groups regarding treatment effects. When performing the equivalent analysis regarding MCS no differences between groups were found (p = 0.136) vs (p = 0.753) (Table 3).

### Physical examination

Subjects younger than 40 years of age in the training group showed improved muscle strength both in the plantar flexors and dorsiflexors at both follow-ups compared to the control group. When including the variables age-group, gender, treatment group and follow-up time in the mixed model analysis there was a significant difference regarding treatment effect in favour of the training group in the plantar flexors (p = 0.007). When also including the interaction of age-group and treatment effect there was a significant difference in favour of the training group in subjects younger than 40 years of age (p = 0.029). After performing the equivalent analyses regarding muscle strength in the dorsiflexors significant differences were found only when interaction of age-group and treatment effect were included in the model (p = 0.030) vs not included (p = 0.145). The timed walking test on even ground and on stairs did not show any significant differences between groups (Table 4).

**Table 4:** Physical outcome 6 and 12 months after surgically treated anklefractures in patient allocated to training group or control group.

**Variables**	**6-month** **Training group**	**6-month** **Usual care group**	**12-month** **Training group**	**12-month** **Usual care group**	**p-value#**
**Dorsal extension injured side (degrees)**					0.752
- all	28 (6.5)	27 (6.6)	30 (6.1)	29 (7.1)	
- <40 years	30 (5.7)	30 (6.5)	33 (5.7)	32 (6.6)	
- >= 40 years	26 (6.4)	26 (6.3)	28 (5.2)	27 (6.9)	

**Plantar flexion injured side (degrees)**					0.736
- all	44 (6.3)	42 (6.6)	47 (6.1)	45 (5.7)	
- <40 years	45 (5.9)	43 (7.0)	48 (5.9)	47 (6.0)	
- >= 40 years	43 (6.4)	41 (6.3)	46 (6.2)	44 (5.1)	

**Rising on toes injured side (n)**					**0.029**
- all	25 (11.8)	19 (10.6)	27 (12.6)	24 (9.0)	
- <40 years	32 (7.4)	21 (11.3)	34 (9.6)	26 (10.6)	
- >= 40 years	19 (11.5)	18 (10.1)	23 (13)	22 (7.0)	

**Rising on heels injured side (n)**					**0.03**
- all	18 (12.9)	16 (12.1)	20 (14.5)	17 (13.2)	
- <40 years	24 (12.4)	15 (10.7)	26 (13.8)	18 (16.5)	
- >= 40 years	14 (11.7)	17 (13.1)	14 (13.8)	11 (10.7)	

**Walking 9 m fast speed (seconds)**					0.084
- all	5 (1.3)	5 (1.1)	4.4 (1.0)	4.6 (1.0)	
- <40 years	4 (0.7)	5 (0.6)	3.9 (0.6)	4.3 (0.9)	
- >= 40 years	5 (1.5)	5 (1.3)	4.8 (1.0)	4.8 (1.1)	

**Walking 12 steps upstairs (seconds)**					0.072
- all	5 (2.2)	6 (1.7)	4.5 (1.7)	4.6 (1.3)	
- <40 years	4 (1.2)	5 (1.7)	3.6 (0.8)	4.0 (1.0)	
- >= 40 years	6 (2.4)	6 (1.7)	5.1 (1.8)	5.0 (1.4)	

#Mixed model analyses on repeated measures for differences between training group and control group and change over time adjusting for gender, age-group, follow-up time and interaction between age-group and treatment effect. The p-values reflect the interaction between age-group and treatment effect.

### Radiographic examination at 12-month follow-up

In all, 98 patients attended at the radiological examination at 12-month. All fractures were healed and 94/98 showed complete joint congruency, < 2 mm in-congruency in one and > 2 mm in-congruency in three cases. Fracture reduction was complete in 65/98 ankles, < 2 mm in-complete in 16/98 ankles and > 2 mm in-complete in 17/98. Nine patients had discrete signs of osteoarthritis (a loss of joint space less than 50%) and two had moderate osteoarthritis (a loss of joint space of more than 50% but no bone-to-bone contact). The results are presented in table by allocation groups (Table 5).

**Table 5:** Radiological examination in patients 12 months after surgically treated ankle fracture

**Variables**	**Training group**	**Control group**	**p-value#**
	**n = 50**	**n = 55**	
**Joint congruency at 12-month**			0.138
- complete	47	47	
- < 2 mm in-congruency	1	0	
- > 2 mm in-congruency	0	3	
- missing	2	5	

**Fracture reduction results at 12-month**			0.085
- complete	37	28	
- < 2 mm displacement	5	11	
- > 2 mm displacement	6	11	
- missing	2	5	

**Osteoarthritis at 12-month**			0.536
- none	41	46	
- <50% loss of joint space	6	3	
- >50% loss of joint space	1	1	
- missing	2	5	

# Chi-square test

## Discussion

The standardised supervised training program employed in this study showed improved results for the exercise group compared to usual care when adjusting for the interaction between age-group and treatment effect. These results concerned both subjectively scored function and clinical physical outcome.

To our knowledge, this is the first randomised controlled trial in patients with surgically treated ankle fractures comparing a standardised and supervised training program with usual care. The strength of this study is the thorough description of the studied groups and the rehabilitation program, the definitions of the physical goals that should be attained and adherence to the randomisation group. Furthermore symptoms, subjectively scored function, clinical physical outcome were evaluated, radiological examination was performed and patients were followed up at both six and 12 months. Patients trained at their nearby primary care physiotherapist therefore the results can be generalised to primary care. A weakness is that patients in the control group could have trained similar exercises as the intervention group. This might have contributed to the small differences between the two groups. Although, the training program designed for the intervention group was not disclosed for control group the physiotherapists that treated the patients in the control group might have practised similar exercises. A reason for this maybe, that the neuromuscular training model is not unfamiliar to physiotherapists in the region. However, the early commencement of training in the intervention group, the structured program and progress of the training program including home exercises and the functional goals that were set up were probably not applied in the control group. Another weakness is that more patients in the control group dropped out. One reason for this might be the lack of attention from care-givers between surgery and the first follow-up at 6-month.

Many studies have evaluated results after ankle fractures without analysing to what extent patients had trained or obtained physiotherapy in order to regain function [10,12,16,33,36,38]. Only one case-control study was found evaluating functions after a rehabilitation period of ten weeks [8]. In that study ten patients (mean 35 years of age) had performed a training program three times per week focusing on strength-training of the plantar flexors in a specially constructed hydraulic apparatus, balance disk training and walking on a tread mill on a level grade. Patients were examined at one, five and ten weeks post-immobilisation. After the ten-week period strength and fatigue resistance in the plantar flexors, walking speed on level ground and on stairs had returned to the same level as in an uninjured control group [8]. In the present study the equivalent variables showed similar results in subjects younger than 40 years.

In the present study more patients than expected in the control group had visited a physiotherapist. In two earlier studies including patients from the same orthopaedic clinic, not being subjected to interventions, only 60% and 56% had received physiotherapy after plaster removal [17,39]. In the present study 76% of the patients in the control group had received physiotherapy. One reason for this increase might be the obligations from the Ethics Committee to give equal and full information to both groups. The patients in the control group were thereby fully aware of what they were "missing" and as they were free to seek care they made appointments on their own. Furthermore due to the obligations from the Ethics Committee the patients could not be prevented from seeking health care. The study was prolonged for more than three years and it cannot be excluded that the surgeons during this time changed their attitude to physiotherapy and increased their referrals. Thereby the usual care could have been also altered during the period of the study. This tells something about the difficulty in performing clinical trials as designed in this study. Instead of randomising to usual care we could have chosen for example a model of home exercises with few but recurrent instructions given by a physiotherapist. The information from that study design would also have been of great interest.

Despite these methodological issues, there were differences in favour of the training group in patients under the age of 40. Symptoms and subjectively scored function as measured by OMAS differed at both follow-ups with the largest score difference at 12-month. The main goal of rehabilitation is to recover function as soon as possible but also to encourage to preserved function over time. It seems as if this had succeeded at least due to symptoms and subjectively scored function as the training group continued to improve.

Regarding the physical outcomes the greatest differences of clinical interest were found in muscle strength. The younger subjects in the training group had better results both in the plantar flexors and dorsiflexors compared to the control group. The recommended reference value for number of rising on toes is stated to 25 [26] except for women over the age of 50 who can be expected to maintain 20 [27]. Subjects in the training group had to a higher extent reached the norms at 6-month compared to the control group. Other studies applying rehabilitation programs directed only to endurance and strength training of the plantarflexors have come to similar conclusions [8,22,40]. In one study the patients after ten weeks of training had resumed muscle strength compared to a control group in a group of ten subjects (3 male and 7 females) aged mean 35 years [8]. In the study by Stevens et al. 20 subjects (9 males and 11 females) aged mean 39 years applying the same training principles the patients had improved but not resumed the same level of muscle strength as the control group [40]. Sufficient strength in the plantar flexors is important as it has been found as a strong predictor of stair-climbing and walking performance [8].

The PCS of SF-36 differed between the groups in patients under the age of 40 in favour of the training group at the 6-month follow-up but did not show significant differences in the mixed model analysis. It is likely to believe that the questions in SF-36 are too common to be able to catch the problems as good as the more ankle specific questionnaire of OMAS. However there are several studies that have shown poorer results using SF-36. Bhandari et al. followed patients mean 52 years of age after surgically treated ankle fractures [36]. Still two years after injury patients had physical effects showing decreased 'physical function' and 'role physical' compared to US norms. Obremsky et al. applied SF-36 as follow-up four and twenty months post surgery in 20 subjects mean age 53 years and found decreased 'physical function' still twenty months after injury compared to the US norms [35]. In the study by Ponzer et al. not only the physical functioning domains but also 'vitality', 'role emotional' and the 'mental health' scores were below the norms of the Swedish population in 53 subjects mean age 41 year [12]. In the present study no analyses have been performed in relation to the norms of the Swedish population but in the light of the referred studies it seems as if patients can be expected to have limitations regarding at least physical functions still two years after injury.

The training program was designed to re-establish joint mobility, rebuild muscle strength and neuromuscular control and to return to pre-injury activity level. Neuromuscular training has been found to be superior to traditional strength training after knee ligament injuries [41] and has been suggested to be used also in other diagnoses since the training aims to resemble conditions in daily life and physical activities [42]. One problem when designing a training program after ankle fractures is the heterogeneity of the patient group. This type of injury is not a typical sports injury but in stead an injury that appears during daily activities and in all ages. Therefore the training program had to be applied as the patient tolerated and in relation to their own expressed functional goals. If there for example was no need for running or jumping in daily life these exercises could be excluded. These routines are normally applied when rehabilitating patients in clinics and the most important guidance in the training progress are the goals established by patient and physiotherapist together. Why only the younger persons had advantage of the training program as designed in the present study is unclear and needs to be further explored. It is possible that older persons need longer time to recover function and need to train for a longer time period or need other types of exercises. Variables like motivation and self-efficacy known to influence functional recovery has not been evaluated in this study but should also be taken into consideration [43]. There are however earlier studies that have come to the same conclusion. Egol et al. reported age under 40 as a predictor of recovery as measured with SMFA subscores (Short Musculoskeletal Function Assessment) at six months after an ankle fracture [16]. All subjects in that study were referred to a physiotherapist, although the training program or adherence was not presented. Furthermore improved results as measured by OMAS have been reported in patients under the age of 40 compared to those over 40 at both one and three years after injury. In that study 60% of the patients had visited a physiotherapist [15].

## Conclusion

The training model used in this study showed superior results compared to usual care regarding subjectively scored function and muscle strength in the plantar flexors and dorsiflexors in patients under the age of 40. However, as only three out of nine outcome measures showed a difference, the beneficial effect from an additional standardised individually suited training program can be expected to be limited. There is need for further studies to elucidate how a training program should be designed to increase and optimise function in patients middle-aged or older.

## Competing interests

The authors declare that they have no competing interests.

## Authors' contributions

GN participated in the design of the study, participated in collecting the data, performed the statistical analyses, and drafted the manuscript. KJ examined all the radiographs. CE participated in the design of the study, and in the progress and revision of the manuscript. ME was responsible for the identifying and including the patients, and participated in the progress and revision of the manuscript. All authors read and approved the final manuscript.

## Pre-publication history

The pre-publication history for this paper can be accessed here:



## Supplementary Material

Additional file 1 **Description of the rehabilitation program**. Training program in patients with surgically treated ankle fractures starting within one week after plaster removal. Click here for file
