# Supplementary material for: Effects of a training program after surgically treated ankle fracture: a prospective randomised controlled trial
Source: BMC Musculoskelet Disord. 2009 Sep 25;10:118. doi: 10.1186/1471-2474-10-118 (PMC2760502; doi:10.1186/1471-2474-10-118)
Supplement: Additional file 1 — Description of the rehabilitation program. Training program in patients with surgically treated ankle fractures starting within one week after plaster removal. [file 1471-2474-10-118-S1.DOC]

**Appendix**

**Training program in patients with surgically treated ankle fractures starting within one week after plaster removal.**

The training program starts within one week after plaster removal and will go on for twelve weeks, two times per week supervised by a physiotherapist and each session lasted one hour. After each session patients should be provided with 2 or 3 home exercises appropriate to functional status at the time. These exercises should be performed daily between sessions. In the program described below only new exercises that are introduced each period are described. The weekly directions are approximate; patient’s recovered function has to be taken into consideration. As long as one function is not regained sufficiently it has to be continued.

**During the whole training period: Be observant of increased pain, swelling or if the ankle gets warm, this could be a sign of too heavy training/loading. Reduce the intensity for one or two weeks.**

*Main goal:*

To provide conditions to the patient so that pre-injury functional level can be regained.

Goals of the rehabilitation process:

- By recurrent and adequate information about the injury, healing process and the importance of gradually increased activities diminish eventual anxiety and fear
- To give recurrent information about the purpose and goal of training and the importance of performed home-exercises (diary can be used)
- Diminish pain and swelling (if a tendency to become swollen, the foot should be elevated when sitting)
- Restore foot and ankle joint mobility, coordination, balance and muscle strength and

endurance

- Retrain normal/pre-injury walking pattern
- Retrain aerobic capacity
- Retrain functions like jumping, running, climbing, rapid turns or other functions required

Goals of physical outcome:

- Dorsal extension 30°
- Plantar flexion 45°
- One leg stance on level ground for 60 seconds
- Rising on toes, one leg, 25; women over the age of 50 should perform 20
- Rising on heels, one leg, 15-20
- Walking on level ground with normalised walking pattern
- Walking upstairs and downstairs with normalised walking pattern
- Walking speed on level ground 15 x 2 metres 18-20 seconds

*Week 1 and 2 (after plaster removal)*:

The exercises during the first one or two weeks are performed only part-loading the foot (in order to avoid iatrogenic injury). All exercises should be performed bilateral in order to bring information to the physiotherapist and feed-back to the patient about the functions of the un-injured side. Crutches are used with weight-bearing as tolerated

- Range of motion training of the ankle in all directions, subtalar joints, toes
- Circulation training by repeated ankle plantar and dorsiflexion range of motion exercises
- Sitting on a stool, training the plantar foot muscles and toe flexors by towel crunches
- Plantar flexors: thera-band as resistance; sitting on a stool, heel-rise with weight on the lap as resistance
- Dorsiflexors: thera-band
- Sit ups
- Training of hip abductors (open chain), hip adductors (press a ball), hip extensors (using cube or large ball as support to the calf), knee extensors (leaning towards a wall slide down)
- Stretching plantar flexors initially by the physiotherapist and later by patient standing on the floor, toe-flexors, hamstrings, quadriceps

*Week 3 and 4:*

Crutches should be discontinued. Weight-bearing exercises are started as soon as possible. Shoes with good support in the longitudinal arch should be used when the foot is loaded in body upright positions.

- Sitting on a stool sliding the foot under the stool with the whole sole of the foot in contact with the floor
- Standing body awareness exercises by moving the centre of pressure of the body forward and laterally (necessary in normal walking).
- Rising on toes and heels, two feet
- Correct walking pattern
- Walking exercises, backward, sideways, zigzag, high knees
- Stationary bicycling low load
- Wobble board two legs
- Step-up forward-backward

*Week 5-6*

If ankle joint mobility not recovered, passive mobility techniques can be incorporated. Be aware of restoring the subtalar joint mobility as well. When performing sliding exercises on the floor or squatting exercises a wedge under the heel may be needed until normal ankle dorsiflexion in the ankle is regained

- Wobble board one leg
- Rising on toes and heels one foot, concentric, excentric
- Walking on toes, walking on heels
- Lunge exercises forward, lateral
- From sitting to standing on one leg
- Walking on a trampoline
- Balance training on trampoline increased difficulties
- Step-up side-way
- Stairs up and down forwards and laterally.
- Stationary bicycling in order to regain aerobic capacity

*Week 7-8*

- Walking up-stairs and down-stairs correctly
- Squatting standing on toes
- Stepping from stool to trampoline
- Jogging on a trampoline
- Sliding exercises laterally and backwards using an sloping board
- Starters with hands towards a wall or gymnasium ribs
- Step-up and down in all directions

*Week 9-12*

During these weeks specific functional activities that the patient wants to return to should be trained. Before running and jumping the walking pattern should be normalised (without limping).

Patient’s functional goals have to decide to whether these exercises should be trained

- Wobble board one leg gradually increased difficulties, weights, throwing a ball, turning the head from side to side
- Lunge exercises with weight
- Jumping exercises, two feet, from one foot to the other, on one foot in different directions
- Running
- Starters from floor
- Preparing for sports

Pictures of the exercises being recommended in this study can be found in [www.mobilus.se](http://www.mobilus.se/) (the English version is under development)
